# Statistical properties of four effect-size measures for mediation models

**DOI:** 10.3758/s13428-017-0870-1

**Published:** 2017-03-24

**Authors:** Milica Miočević, Holly P. O’Rourke, David P. MacKinnon, Hendricks C. Brown

**Affiliations:** 1 Department of Psychology, Arizona State University, 950 S. McAllister Ave, Tempe, AZ 85287 USA; 2 Feinberg School of Medicine, Northwestern University, Chicago, IL USA

**Keywords:** Mediation, Effect sizes, Bias, Efficiency, Interval estimates, Bayesian methods

## Abstract

This project examined the performance of classical and Bayesian estimators of four effect size measures for the indirect effect in a single-mediator model and a two-mediator model. Compared to the *proportion* and *ratio* mediation effect sizes, standardized mediation effect-size measures were relatively unbiased and efficient in the single-mediator model and the two-mediator model. Percentile and bias-corrected bootstrap interval estimates of *ab*/*s*_*Y*_, and *ab*(*s*_*X*_)/*s*_*Y*_ in the single-mediator model outperformed interval estimates of the *proportion* and *ratio* effect sizes in terms of power, Type I error rate, coverage, imbalance, and interval width. For the two-mediator model, standardized effect-size measures were superior to the *proportion* and *ratio* effect-size measures. Furthermore, it was found that Bayesian point and interval summaries of posterior distributions of standardized effect-size measures reduced excessive relative bias for certain parameter combinations. The standardized effect-size measures are the best effect-size measures for quantifying mediated effects.

## Introduction

This research addresses three developing trends in social sciences research: (1) the increasingly frequent use of mediation models; (2) the growing awareness of the importance of effect sizes for scientific progress, as shown by scientific journals now demanding that researchers report effect-size measures in their articles (Wilkinson & American Psychological Association Task Force on Statistical Inference, 1999; Vacha-Haase & Thompson, 2004); and (3) the increased interest in Bayesian methods. Despite these trends, there is still no preferred effect-size measure for the indirect effect, and Bayesian estimation of effect-size measures has not been described for mediation models. The first purpose of this article is to investigate the statistical properties of two effect-size measures that have not been investigated extensively in simulation studies thus far, and assess their performance compared with the two most frequently used effect-size measures in the substantive literature. The second purpose of this article is to describe the computation and interpretation of Bayesian point and interval summaries of effect-size measures in mediation models, and to probe their frequentist properties in a simulation study.

### Effect-size measures for mediation models

Mediation analysis is conducted to understand the mechanisms through which one variable influences another. The simplest case of mediation is the single-mediator model, where an independent variable X affects a dependent variable Y though a mediator M. This single-mediator model with independent observations can be described using three equations:

1 $$ \mathrm{Y}={i}_1+ c\mathrm{X}+{e}_1 $$

2 $$ \mathrm{Y}={i}_2+ c\hbox{'}\mathrm{X}+ b\mathrm{M}+{e}_2 $$

3 $$ \mathrm{M}={i}_3+ a\mathrm{X}+{e}_3 $$

and estimated using only equations 2 and 3, or only equations 1 and 3 (MacKinnon, 2008). There are two equivalent ways to compute the mediation (indirect) effect in the above model when M and Y are continuous and the model is estimated using ordinary least squares regression or Maximum Likelihood (ML) estimation. One can obtain the product of the coefficients *a* and *b*, or subtract the direct effect from the total effect *c−c’*. Most formulas for effect-size measures for the indirect effect use the product of coefficients, *ab*, since computing the mediated effect this way can easily be extended to models with multiple mediators.

Adding a second mediator to the model will result in the following equations:

4 $$ \mathrm{Y}={i}_1+ c\mathrm{X}+{e}_1 $$

5 $$ \mathrm{Y}={i}_2+ c\hbox{'}\mathrm{X}+{b}_1{\mathrm{M}}_1+{b}_2{\mathrm{M}}_2+{e}_2 $$

6 $$ {\mathrm{M}}_1={i}_3+{a}_1\mathrm{X}+{e}_3 $$

7 $$ {\mathrm{M}}_2={i}_4+{a}_2\mathrm{X}+{e}_4 $$

where each mediator is predicted by X because each mediator is intermediate between the X and Y variables. The two mediators, M_1_ and M_2_, are usually permitted to co-vary. This model is called the parallel two-mediator model, and the indirect effect is represented as either *a*_*1*_ *b*_*1*_ *+a*_*2*_ *b*_*2*_ or *c−c’*. The total indirect effect of interest here is *a*_*1*_ *b*_*1*_ *+a*_*2*_ *b*_*2.*_

If the independent and dependent variables have clear units of measurement, e.g., pounds or words, then reporting the indirect effect has a meaningful interpretation in terms of pounds or words. If X and Y do not have intuitive units of measurement, it is a good idea to report at least one effect-size measure. Effect sizes for mediation are divided into effect sizes for the individual paths in the indirect effect and effect sizes for the overall indirect effect (MacKinnon, 2008). Depending on the researcher’s substantive questions, there are multiple effect-size measures for the single-mediator model one can choose from (MacKinnon, 2008; Preacher & Kelley, 2011). If the study focuses on action theory (theory underlying the *a* path) and conceptual theory (theory underlying the *b* path), it may be of interest to report the correlation or standardized regression coefficients for the *a* and *b* paths, respectively. There are more options for reporting the effect size of the indirect effect, and the ideal measure depends on the substantive question. For example, the researcher could be interested in reporting the portion of variance in Y explained by the indirect effect (Fairchild, MacKinnon, Taborga, & Taylor, 2009). One might also be interested in reporting the proportion of the effect of X on Y that is mediated, and thus might opt for *proportion* mediated as an effect-size measure. Similarly, the focus of the study might be the comparison of the magnitudes of the indirect and direct effects, and the ideal effect size would be the *ratio* mediated. One could also choose to standardize the indirect effect by the standard deviation of the dependent variable alone, or by the standard deviations of both the independent and dependent variables (Cheung, 2009; MacKinnon, 2008). Recently, effect-size measures have been proposed that quantify the maximum possible mediation effect that could have been achieved given the constraints of the data and the proportion of that maximum effect that was obtained in a given study (Preacher & Kelley, 2011). However, these effect-size measures have been criticized for their lack of rank preservation and were not recommended for use in any set of models, including mediation models (Wen & Fan, 2015).

### Reasons for the study

There have been many mediation effect-size measures proposed, as well as multiple ways to define the meaning of an effect size in a mediation model (MacKinnon, 2008; Preacher & Kelley, 2011). The statistical properties of the *proportion* and *ratio* mediated (Freedman, 2001; MacKinnon, Warsi, & Dwyer, 1995), *ab(s*_*X*_ *)/s*_*Y*_ (Cheung, 2009), and R^2^ (Fairchild, MacKinnon, Taborga, & Taylor, 2009) have been studied in simulation studies (Taborga, 2000); however, this is not the case with *ab/s*_*Y*_ for the single-mediator model, and (*a*_*1*_ *b*_*1*_ *+a*_*2*_ *b*_*2*_ *)/s*_*Y*_, (*a*_*1*_ *b*_*1*_ *+a*_*2*_ *b*_*2*_ *)(s*_*X*_ *)/s*_*Y*_, the *proportion*, and the *ratio* mediated for the two-mediator model. It is known that standardized regression coefficients are unbiased with sample sizes of at least 50 (Yuan & Chan, 2011), that bootstrap intervals for unstandardized *ab* (Mackinnon, Lockwood, & Williams, 2004), and *ab(s*_*X*_ *)/s*_*Y*_ have coverage close to nominal value (Cheung, 2007; 2009), and that the *proportion* mediated requires large sample sizes, large effects, or both in order to have unbiased point estimates and standard errors (Freedman, 2001; MacKinnon, Warsi, & Dwyer, 1995). However, *ab(s*_*X*_ *)/s*_*Y*_ is seldom reported by substantive researchers, and the *proportion* mediated is still the most frequently reported effect-size measure for mediation models, followed closely by the *ratio* mediated. An online search with Google Scholar and PsycInfo with the keywords “mediation, mediated effect, proportion mediated, ratio mediated, standardized mediated effect” was conducted in order to determine which effect-size measures are reported most often. The vast majority of studies that report a mediation analysis do not report an effect size for the mediated effect, and out of all possible effect-size measures for the indirect effect, only *ratio* and *proportion* mediated seem to appear in the substantive literature (Barreto & Ellemers, 2005; Chassin, Pitts, DeLucia, & Todd, 1999; Ilies & Judge, 2003; Ilies & Judge, 2005; Leigh, 1983; MacKinnon, Johnson, Pentz, Dwyer, Hansen, Flay, & Wang, 1991; Sharkansky, King, King, Wolfe, Erickson, & Stokes, 2000; Stice, 2001; Tein, Sandler, Ayers, & Wolchik, 2006; Wolchik, West, Westover, Sandler, Martin, Lustig, Tein, & Fisher, 1993).

This study will enhance the simulation literature by investigating the statistical properties of *ab/s*_*Y*_ for the single-mediator model, and (*a*_*1*_ *b*_*1*_ *+a*_*2*_ *b*_*2*_ *)/s*_*Y*_, (*a*_*1*_ *b*_*1*_ *+a*_*2*_ *b*_*2*_ *)(s*_*X*_ *)/s*_*Y*_, the *proportion*, and the *ratio mediated* for the two-mediator model. Results will inform substantive researchers that have sample sizes smaller than 500 (and thus cannot report the *proportion* or *ratio* mediated without risking bias and instability) about alternative effect-size measures of the indirect effect that are unbiased, efficient, have intervals with desirable statistical properties, and offer intuitive interpretations.

### The focus of the studies

The first goal of the Monte Carlo studies in this article is to determine which effect-size measures for the indirect effect out of four candidates (the partially standardized mediated effect, the fully standardized mediated effect, the *proportion* mediated, and the *ratio* mediated) have point estimates with low bias and high stability for a variety of combinations of parameter values and sample sizes. The second goal is to evaluate the interval properties (i.e., Type I error rate, power, coverage, interval width, and imbalance) of two methods for constructing confidence intervals for the same four effect-size measures. The first two goals focus on classical (frequentist) methods for parameter estimation. The third goal of this article is to describe Bayesian methods as an alternative to classical methods for effect size computation, and to subsequently probe the statistical properties of Bayesian methods for computing the four effect-size measures.

The partially standardized indirect effects for the single and two-mediator models are as follows:

8 $$ a{b}_{ps}= ab/{s}_Y $$

9 $$ a{b}_{ps}=\left({a}_1{b}_1+{a}_2{b}_2\right)/{s}_Y $$

These effect sizes capture the size of the indirect effect in terms of standard deviations of the dependent variable for a one unit change in the independent variable. Instead of dividing the indirect effect by the standard deviation of the dependent variable Y, one could compute this effect-size measure by first standardizing the dependent variable Y and simply computing the indirect effect *ab*; the numerical value and interpretation of this quantity would remain the same. When X is a binary grouping variable, the indirect effect is in terms of change in standard deviation units of Y between the two groups, making this effect-size measure ideal for the case where X represents randomization to one of two conditions.

Another standardized effect-size measure for the indirect effect is the fully standardized indirect effect:

10 $$ a{b}_{fs}= ab\left({s}_X\right)/{s}_Y $$

11 $$ a{b}_{fs}=\left({a}_1{b}_1+{a}_2{b}_2\right)\left({s}_X\right)/{s}_Y $$

These effect-size measures give the magnitude of the indirect effect in standard deviations of both the independent and the dependent variables for a one standard deviation increase in the independent variable. It is the effect of a standard deviation increase in X on standard deviation units of Y.

Two effect sizes assessing the relative magnitude of the indirect effect will also be compared. The *proportion* mediated is calculated by dividing the indirect effect by the total effect for the single-mediator model (12) and for the two-mediator model (13):

12 $$ proportion= ab/\left( ab+ c'\right) $$

13 $$ proportion=\left({a}_1{b}_1+{a}_2{b}_2\right)/\left({a}_1{b}_1+{a}_2{b}_2+ c'\right) $$

This effect size is useful when one is interested in the proportion of the total effect that is due to the indirect effect. What seems like a small indirect effect might be relatively large when compared to the total or direct effects, and conversely, a seemingly large indirect effect might seem small once compared to the total and direct effects. The interpretation of *proportion* mediated is somewhat complicated in inconsistent mediation models (when the direct and indirect effects are of opposite signs), and further complicated by the addition of multiple mediators. Another way to quantify the importance of the mediation effect is to calculate the ratio of the indirect effect to the direct effect. To do so, the indirect effect is divided by the direct effect (MacKinnon, 2008):

14 $$ ratio= ab/ c' $$

15 $$ ratio=\left({a}_1{b}_1+{a}_2{b}_2\right)/ c' $$

Equation 12 is the formula for the *ratio* mediated for the single-mediator model and equation 13 is the formula for the *ratio* mediated for the two-mediator model. The *proportion* and *ratio* mediated can be computed for any mediation model; however, they are not appropriate measures of effect size when complete mediation is present (that is, *c’* is equal to zero). In this case, the *proportion* mediated is equal to one and the *ratio* mediated is undefined. Furthermore, prior research shows that the *proportion* and *ratio* mediated require large sample sizes (500 and 5,000 if X in binary, respectively) in order to have unbiased point estimates and standard errors (MacKinnon, Warsi & Dwyer, 1995). Despite these issues, the *proportion* and *ratio* mediated will be included in this study because they are the two effect-size measures for the indirect effect most commonly encountered in the substantive literature, which makes them a good reference point for evaluating statistical properties of the remaining effect-size measures tested in this study.

In addition to computing a point estimate, it is important to compute an interval estimate of the effect size in order to quantify the uncertainty about the estimate (Feingold, 2014; Kelley, 2005; Kraemer, 2014; Stapleton, Pituch & Dion, 2015; Wilkinson & American Psychological Association Task Force on Statistical Inference, 1999). The goal of the second Monte Carlo study is to examine the statistical properties of interval estimators of effect-size measures for the mediated effect. The fact that the distribution of the product of two normal quantities is not normal has been well-documented (Craig, 1936; Lomnicki, 1967; Springer & Thompson, 1966), and for this reason methods outside of normal theory have been used to construct interval estimates of the indirect effect (MacKinnon, Lockwood, Hoffman, West & Sheets, 2002; MacKinnon, Lockwood, and Williams, 2004). The distributions of effect-size measures for the indirect effect are unknown, but given that the indirect effect is one of the terms in their computation, they are likely not normal. Thus normal theory would not be ideal for computing interval estimates of effect-size measures for the indirect effect.

One common non-parametric alternative to normal theory is the bootstrap (Manly, 1997; MacKinnon, Lockwood, & Williams, 2004; MacKinnon, 2008; Shrout & Bolger, 2002). Bootstrap methods consist of resampling the observed data in order to construct a distribution of the estimate of interest. Once the estimate of interest has been calculated from the desired number of samples, a confidence interval for the estimate can be formed from the α/2 and (1- α/2) points of the distribution. In the case of an effect size for the indirect effect, bootstrapping would consist of sampling N observations with replacement from the original sample of size N, calculating the effect size, and repeating this procedure a large number of times. After a large number of iterations a distribution of the effect size has been formed, and the 95% confidence interval is constructed from the 2.5% and 97.5% quantiles of the distribution. This method is called the percentile bootstrap or Efron’s percentile method.

The percentile bootstrap assumes the existence of a transformation that preserves the order of the parameter of interest. However, such a transformation may not always exist, and bias arises when the true value of the parameter does not correspond to the median of the distribution of estimates (Manly, 1997). Bias is handled by finding the proportion of times *p* that the bootstrapped estimates exceed the sample (observed) value of the estimate, and z_0_ which is the z value that corresponds to this *p-*value. This method is called the bias-corrected percentile bootstrap. The lower confidence limit is then the estimate that just exceeds the proportion *φ*(2*z*_0_ + *z*_*α*/2_) of all values in the bootstrap distribution of estimates. The upper confidence limit for the estimate is the value that exceeds a proportion *φ*(2*z*_0_ + *z*_1 − *α*/2_) in that same distribution (Manly, 1997). Both the percentile and the bias-corrected bootstrap methods have been found to work well in the construction of intervals for the indirect effect (Biesanz, Falk, & Savalei, 2010; Cheung, 2007; MacKinnon, Lockwood & Williams, 2004). For this reason, and because they do not require distributional assumptions, the percentile and bias-corrected bootstrap are used to construct intervals for effect-size measures for the indirect effect. Furthermore, there has been a recent increase in the use of Bayesian methods in social sciences due to the possibility of estimating models with smaller sample sizes than those needed for maximum likelihood estimation (Lee & Song, 2004), and because of the probabilistic interpretations of parameters (van de Schoot & Depaoli, 2014). Like any other parameter, effect-size measures can be computed in the Bayesian framework. Bayesian methods with diffuse priors have been found to produce intervals with satisfactory statistical properties for the indirect effect (Miočević, MacKinnon, & Levy, 2016), but have yet to be used for computing effect-size measures for the mediated effect.

The examination of effect-size measures for the single and parallel two-mediator models will proceed in several Monte Carlo studies focusing on the statistical properties of point and interval estimates, and the potential of Bayesian methods for effect size computation. For criteria other than statistical properties examined in this study that might influence the selection of the effect-size measure for the indirect effect, see Preacher and Kelley (2011) and MacKinnon (2008). The first set of Monte Carlo studies in this project are evaluating classical (frequentist) methods for computing effect-size measures for the indirect effect. The second half of this article describes Bayesian methods for effect size computation and uses a Monte Carlo study to examine the frequentist properties of Bayesian point and interval summaries of effect-size measures on a small set of parameter combinations.

## Monte Carlo studies of classical estimators

Several Monte Carlo studies were conducted in order to evaluate the statistical properties of classical (frequentist) point and interval estimates of the four effect-size measures for the mediated effect in the single-mediator model and the two-mediator model. The purpose of the first Monte Carlo study was to assess the bias and efficiency of frequentist point estimates of effect-size measures for the single and two-mediator models. Bias was defined as the difference between the estimate and the population value of the effect size. Efficiency was defined as the change in the value of an effect size from one sample to another. It should be noted that MSE could also be used as a measure of efficiency, as was done by Krull and MacKinnon (1999). However, since bias is already an outcome measure in this study, it was of interest to have an outcome measure that only captures the variability of the effect-size measures over repeated sampling. Therefore, in this article a smaller value of the standard deviation (i.e., smaller changes in the value of an effect size from one sample to the next) corresponded to greater efficiency. Monte Carlo studies were also used to evaluate frequentist interval estimators of effect-size measures for the single and parallel two-mediator models. Example SAS code for all Monte Carlo studies in this manuscript is available online at https://figshare.com/s/8d48fed4a23fff78e2a3 .

## Methods

### Single-mediator model

In a study containing 320 combinations of parameters, SAS software (Version 9.2 of the SAS System for Windows, Cary, NC, USA) was used to conduct a simulation which calculated bias, relative bias, and standard deviations of effect sizes over 1,000 replications. In the first simulation, all three variables (X, M, and Y) were continuous, and the variance of X and residual variances of M and Y were simulated with a value of 1. A macro was designed to loop through all combinations of sample sizes (10, 50, 100, 500, and 1,000) and population values for *a*, *b*, and *c’* paths (0, 0.14, 0.39, and 0.59). The population values of paths *a*, *b*, and *c’* have been chosen to correspond to approximately zero, small (2% of the variance), medium (13% of the variance), and large (26% of the variance) effect sizes as described by Cohen (1988). Means of bias, relative bias, and the standard deviations were obtained for each combination over 1,000 replications.

Bias is not an ideal measure because it is affected by the value of the effect-size measure; that is, bias will be smaller only because the numerical size of the measure is smaller. A better measure is relative bias which scales bias by the true value of the effect (Krull & MacKinnon, 1999). Relative bias is defined only for combinations that have non-zero paths for the true effect. This study used the value of .05 as a cut-off value for relative bias (half of .10, the value used by Kaplan (1988)), and all values with relative bias above .05 were considered problematic. The standard deviation of the estimate over replications was a measure of efficiency, where higher standard deviation indicated a less efficient estimator. Standardized bias was subsequently computed from the output of the simulation by dividing the values of bias by the standard deviations corresponding to the same combination of parameter values and sample size. When comparing the standardized bias of two effect-size measures, larger values of standardized bias indicate that one effect-size measure has more raw bias relative to its standard deviation than another effect-size measure. Comparable values of standardized bias for two effect-size measures indicate that the values of raw bias of the two effect-size measures increase in comparable amounts with increases in their inefficiency. In the second simulation, M and Y remained continuous, while X was a binary variable. The same parameter values were used in both simulations, and the analyses from the first simulation were repeated at the end of the second simulation.

The third and fourth simulations were designed to calculate the empirical power, interval width, coverage, empirical Type I error rate, and imbalance of percentile bootstrap and bias-corrected bootstrap interval estimates of *ab/s*_*Y*_, *ab(s*_*X*_ *)/s*_*Y*_, the *proportion* mediated, and the *ratio* mediated for the single-mediator model with continuous and binary X. A macro was designed to loop through all combinations of sample sizes (50_,_ 100, 500, and 1,000) and population values for *a*, *b*, and *c’* paths (0, 0.14, 0.39, and 0.59). Empirical power was defined as the percentage of confidence intervals for the effect-size measure that did not contain zero when a true effect exists in the population; values of 0.8 and higher were deemed desirable. Interval width was defined as the difference between the upper confidence limit and the lower confidence limit; smaller interval width indicates more precision of the estimate, however, since the four effect-size measures are not on the same metric their interval widths cannot be directly compared, thus decreasing interval widths with increases in sample size was used as a criterion. Coverage was defined as the proportion of confidence intervals that contained the true value of the effect-size measure; in this study coverage closest to 0.95 was deemed desirable. The empirical Type I error rate was the percentage of confidence intervals that did not contain zero when the true value of the effect-size measure in the population was zero; a Type I error rate of .05 was the nominal level in this study. Imbalance was defined as the disparity between the true values that fall on the right side of the confidence interval versus on the left side of it; imbalance closer to zero was more desirable. In one of the simulations, X, M, and Y were continuous, and in the other simulation X was binary. Type I error rate and coverage were evaluated using Bradley’s robustness criterion (1978), thus values of Type I error rate between 0.025 and 0.075 were deemed appropriate, and values of coverage between 0.925 and 0.975 were deemed close to nominal level.

### Two-mediator model

The simulations for the two-mediator model with continuous X and binary X were conducted in a similar fashion as the simulations for the single-mediator model. A macro was designed to loop through different combinations of sample sizes (10, 50, 100, and 500) and path parameters (for *a*_*1*_, *b*_*1*_, *a*_*2*_, and *b*_*2*_: 0, 0.101, 0.314, and 0.577; for *c’*: 0, 0.131, 0.400, and 0.740) for each of the *a*_*1*_, *a*_*2*_, *b*_*1*_, *b*_*2*_, and *c’* paths. The population values of paths *a*, *b*, and *c’* were chosen to correspond to approximately zero, small (2% of the variance), medium (13% of the variance), and large (26% of the variance) effect sizes as described by Cohen (1988), and previously used in simulation work by O’Rourke and MacKinnon (2015). As the inclusion of additional paths in the two-mediator model substantially increases the number of possible combinations of parameters and sample sizes, only those combinations where *a*_*1*_=*b*_*1*_ and *a*_*2*_=*b*_*2*_ were used for this study, leading to 256 combinations. Means of bias and relative bias, and the standard deviations were obtained for each combination over 1,000 replications, with standardized bias calculated from the simulation results.

An additional simulation was conducted to calculate empirical power, interval width, coverage, empirical Type I error rate, and imbalance of percentile bootstrap and bias-corrected bootstrap interval estimates of *ab*_*ps*_, *ab*_*fs*_, the *proportion* and the *ratio* mediated for the two-mediator model over 1,000 replications. A macro was designed to loop through different combinations of sample sizes (10, 50, 100, and 500) and path parameters (for *a*_*1*_, *b*_*1*_, *a*_*2*_, and *b*_*2*_: 0, 0.101, 0.314, and 0.577; for *c’*: 0, 0.131, 0.400, and 0.740) for each of the *a*_*1*_, *a*_*2*_, *b*_*1*_, *b*_*2*_, and *c’* paths. Empirical power, interval width, coverage, imbalance, and Type I error rate were defined and calculated as they were for the single-mediator model. In one of the simulations, X, M_1_, M_2_, and Y were continuous, and in the other simulation X was binary. As in the analysis of the single-mediator model, Bradley’s robustness criterion (1978) was used to evaluate Type I error rate and coverage. The results of the analyses are presented below, along with explanations of the findings.

## Results

### Single-mediator model

#### Bias and efficiency

For the single-mediator model with both continuous and binary X, the range of the values of bias for *ab/s*_*Y*_ and *ab(s*_*X*_ *)/s*_*Y*_ decreased as sample size increased from N = 10 to N = 1,000. There was no such trend for the *proportion* and *ratio* mediated. Also, *ab/s*_*Y*_ and *ab(s*_*X*_ *)/s*_*Y*_ showed a monotonic decrease in bias as sample size increased, whereas the *proportion* and *ratio* mediated did not.

**Fig. 1:**
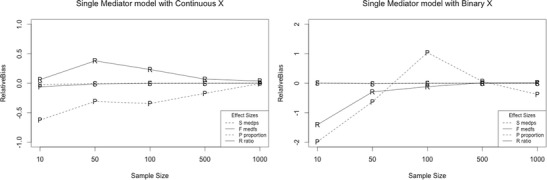
Trellis plot of relative bias for all effect size measures as a function of sample size for the single-mediator model. The letter markers indicate the following: S codes *med*_*ps*_, F codes *med*_*fs*_, P codes the *proportion* mediated, and R codes the *ratio* mediated

Regardless of sample size, *ab/s*_*Y*_ and *ab(s*_*X*_ *)/s*_*Y*_ had noticeably smaller ranges of relative bias than the *proportion* and *ratio* mediated. A decrease in relative bias as sample size increased was observed for *ab/s*_*Y*_ and *ab(sX)/sY* but not for the *proportion* and *ratio* mediated (Fig. 1). For sample sizes smaller than 500, *ab/s*_*Y*_ and *ab(s*_*X*_ *)/s*_*Y*_ had fewer instances of excessive relative bias than the *proportion* and *ratio* mediated. This was also the case for sample sizes of 500 and 1,000 when the direct effect was zero or small. When the direct effect was medium and large and sample size was at least 500, none of the effect-size measures had excessive relative bias.

The efficiency (stability) of effect-size measures was evaluated based on their standard deviations in the 1,000 simulations for each combination of effect size and sample size. As sample size increased, the ranges of standard deviation estimates monotonically decreased for *ab/s*_*Y*_ and *ab(s*_*X*_ *)/s*_*Y*_, but not for the *proportion* and *ratio* mediated, meaning that *ab/s*_*Y*_ and *ab(s*_*X*_ *)/s*_*Y*_ became more stable (efficient) with increased sample size. The *ratio* and *proportion* mediated were comparatively unstable (inefficient) regardless of sample size.

Standardized bias was computed by dividing the bias of an effect-size measure by its standard deviation at each combination of parameter values and sample size. The four effect-size measures had comparable ranges of standardized bias for all sample sizes meaning that bias and efficiency were proportional for all four effect-size measures. In other words, the more biased an effect-size measure, the less efficient it was. Conversely, effect-size measures that had low bias also had small standard deviations.

#### Power

For both continuous and binary X and at all sample sizes, *ab/s*_*Y*_, and *ab(s*_*X*_ *)/s*_*Y*_ had identical values of power that were higher than power values for the *proportion* mediated at *c’*=0 and 0.14, and higher than power for the *ratio* regardless of value of *c’*. The values of power for the *proportion* mediated became closer or equal to values of power for *ab/s*_*Y*_, and *ab(s*_*X*_ *)/s*_*Y*_ when *c’*=0.39 and 0.59 and for larger values of *a* and *b*. Power of the *ratio* mediated started to approach power of the remaining three effect sizes when the direct effect was large and N = 100. Larger sample size and values of *a* and *b* for a given value of *c’* corresponded with smaller differences in power for the percentile bootstrap estimates of *ab/s*_*Y*_, *ab(sX)/sY*, and the *proportion* and *ratio* mediated. With continuous X, the bias-corrected bootstrap estimates of *ab/sY* and *ab(sX)/sY* had the highest power at all sample sizes. The *proportion* mediated had slightly lower power, and the *ratio* mediated had the lowest power of all effect sizes. When X was binary, the bias-corrected bootstrap estimates of the *ratio* and *proportion* mediated had higher power than the other effect-size measures at N = 50, but at larger sample sizes *ab/sY* and *ab(sX)/sY* had more power than the *proportion* and *ratio* mediated. The differences in power between the four effect-size measures for both the percentile and the bias-corrected bootstrap estimates were most pronounced when there was no direct effect *c’* and when *c’*=0.14, and at sample sizes smaller than 500.

#### Type I error rate

The percentile bootstrap estimates of the four effect-size measures never had Type I error rates above 0.075, the upper limit of the robustness criterion. In fact, all effect-size measures had Type I error rate below 0.025 in approximately half of the parameter combinations. For some combinations of parameter values and sample sizes, the bias-corrected bootstrap estimates had Type I error rates above 0.075. This occurred more often for the *proportion* and *ratio* mediated, but occurred less for these effect sizes at larger sample sizes. All effect-size measures had instances of Type I error rate below 0.025 with bias-corrected bootstrap interval estimation. Overall, with continuous and binary X the Type I error rates for the percentile bootstrap estimates of effect sizes were never excessive and were often below 0.025, whereas the bias-corrected bootstrap estimates produced instances of Type I error rate above 0.075.

#### Coverage

The percentile bootstrap interval estimates of *ab/sY*, *ab(sX)/sY*, and the *ratio* mediated had coverage within or above the robustness criterion for all parameter combinations and at all sample sizes examined in this study regardless of whether X was continuous or binary. The *proportion* mediated had a few instances of coverage below 0.925 when sample size was smaller than 1,000.

Coverage below 0.925 was noticeably more prevalent with the bias-corrected bootstrap estimates of effect-size measures. The bias-corrected bootstrap estimates of the *proportion* and *ratio* mediated had more instances of coverage below 0.925 than of *ab/s*_*Y*_ and *ab(sX)/sY*. An important finding is that coverage is far more satisfactory when using the percentile bootstrap than the bias corrected bootstrap for all effect sizes in the single-mediator model with both continuous and binary X.

#### Interval width

**Fig. 2:**
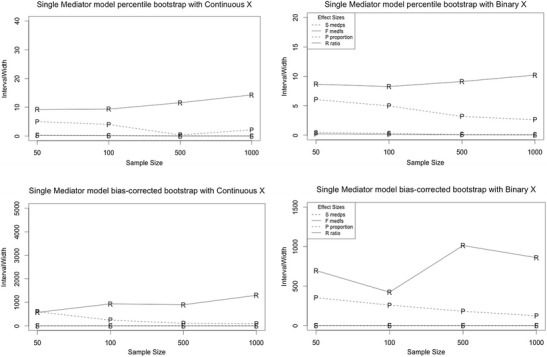
Trellis plot of interval width for percentile and bias-corrected bootstrap estimates of all effect size measures as a function of sample size for the single-mediator model. The letter markers indicate the following: S codes *med*_*ps*_, F codes *med*_*fs*_, P codes the *proportion* mediated, and R codes the *ratio* mediated

Interval widths of percentile and bias-corrected bootstrap estimates of *ab/sY* and *ab(sX)/sY* were consistently lower than interval widths of the *proportion* and *ratio* mediated in models with continuous and binary X (Figs. 2). The discrepancy between the effect-size measures was most noticeable when *c’*=0 and 0.14, and for sample sizes smaller than 500. However, the four effect-size measures are on different metrics, and thus their interval widths cannot be compared. Thus, the criterion of decreasing interval width with increasing sample size (reflecting an increase in precision with increases in sample size) is used to evaluate the effect sizes. The interval width of the *proportion* and *ratio* mediated for a given combination of parameter values did not consistently decrease with increased sample size, meaning that increasing sample size did not guarantee a more precise estimate for these two effect-size measures. The findings indicate that interval estimates of *ab/sY* and *ab(sX)/sY* became more precise as sample size increased.

#### Imbalance

When the direct effect was zero and the *ratio* mediated was undefined, the *proportion* mediated had the highest imbalance of the effect sizes. At non-zero values of *c’*, the four effect-size measures had comparable imbalance for most values of *a* and *b*, and in cases where one effect-size measure had higher imbalance than others, it was most often the *ratio* or the *proportion* mediated. The findings for the bias-corrected bootstrap were similar, and for most combinations of parameter values, imbalance was higher for the bias-corrected bootstrap. Furthermore, *ab/sY* and *ab(sX)/sY* had less imbalance than the *proportion* and *ratio* mediated at small sample sizes and for zero and small values of *c’*.

Overall, the evaluation of Type I error rate, power, coverage, interval width, and imbalance of percentile and bias-corrected bootstrap interval estimates of four effect-size measures for the single-mediator model indicated that *ab/s*_*Y*_ and *ab(s*_*X*_ *)/s*_*Y*_ tended to outperform the *proportion* and *ratio* mediated on the five criteria, and that percentile bootstrap is preferred over bias-corrected bootstrap in terms of Type I error rate, coverage, and imbalance.

### Two-mediator model

#### Bias and efficiency

For the two-mediator model with continuous X, the range of values of bias for (*a*_*1*_ *b*_*1*_ *+a*_*2*_ *b*_*2*_ *)/s*_*Y*_, (*a*_*1*_ *b*_*1*_ *+a*_*2*_ *b*_*2*_ *)(s*_*X*_ *)/s*_*Y*_, and the *ratio* effect sizes systematically decreased as sample size increased from *N* = 10 to *N* = 500, whereas this was not the case for the *proportion* mediated. When X was binary, the range of values of bias decreased as sample size increased only for (*a*_*1*_ *b*_*1*_ *+a*_*2*_ *b*_*2*_ *)/s*_*Y*_ and (*a*_*1*_ *b*_*1*_ *+a*_*2*_ *b*_*2*_ *)(s*_*X*_ *)/s*_*Y*_.

**Fig. 3:**
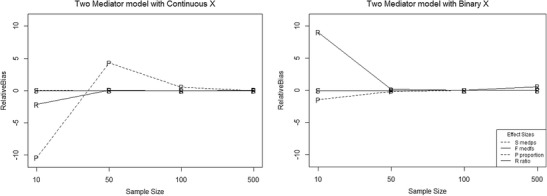
Trellis plot of relative bias for all effect size measures as a function of sample size for the two-mediator model. The letter markers indicate the following: S codes *med*_*ps*_, F codes *med*_*fs*_, P codes the *proportion* mediated, and R codes the *ratio* mediated

The range of relative bias decreased for all four effect-size measures for the two-mediator model with continuous X. When X was binary, the range of relative bias increased with increasing sample size only for the standardized effect-size measures. With continuous X for (*a*_*1*_ *b*_*1*_ *+a*_*2*_ *b*_*2*_ *)/s*_*Y*_ and (*a*_*1*_ *b*_*1*_ *+a*_*2*_ *b*_*2*_ *)(s*_*X*_ *)/s*_*Y*_, relative bias of .05 was exceeded in certain parameter combinations when N=10 or 50 and *a*_*1*_ *=b*_*1*_ and *a*_*2*_ *=b*_*2*_ were zero or small; however, relative bias for these two effect-size measures was below .05 for N = 100, 500 for all values of coefficients. Thus, with continuous X the partially and fully standardized indirect effects were unbiased at N=10 and 50 if the effects were medium or large, and at N=100 and 500 regardless of the size of the effects. In addition, (*a*_*1*_ *b*_*1*_ *+a*_*2*_ *b*_*2*_ *)/s*_*Y*_ and (*a*_*1*_ *b*_*1*_ *+a*_*2*_ *b*_*2*_ *)(s*_*X*_ *)/s*_*Y*_ had much smaller relative bias values than the *proportion* and *ratio* mediated. With binary X, (*a*_*1*_ *b*_*1*_ *+a*_*2*_ *b*_*2*_ *)/s*_*Y*_ and (*a*_*1*_ *b*_*1*_ *+a*_*2*_ *b*_*2*_ *)(s*_*X*_ *)/s*_*Y*_ had smaller relative bias values than the *proportion* and *ratio* mediated, although both (*a*_*1*_ *b*_*1*_ *+a*_*2*_ *b*_*2*_ *)/s*_*Y*_ and (*a*_*1*_ *b*_*1*_ *+a*_*2*_ *b*_*2*_ *)(s*_*X*_ *)/s*_*Y*_ continued to have relative bias values greater than 0.05 even at *N* = 500 (Fig. 3).

For both continuous and binary X standard deviations for (*a*_*1*_ *b*_*1*_ *+a*_*2*_ *b*_*2*_ *)/s*_*Y*_ and (*a*_*1*_ *b*_*1*_ *+a*_*2*_ *b*_*2*_ *)(s*_*X*_ *)/s*_*Y*_ decreased as sample size increased, and were relatively low (never exceeding 0.5). When X was binary, the standard deviations of the *proportion* and *ratio* mediated decreased as *c’* increases, and the *proportion* mediated became more efficient at larger sample sizes, whereas the *ratio* mediated becomes less efficient as N increased.

For standardized bias, the pattern of results for the two-mediator model with continuous and binary X differed from the results found for the single-mediator model. For this model, both (*a*_*1*_ *b*_*1*_ *+a*_*2*_ *b*_*2*_ *)/s*_*Y*_ and (*a*_*1*_ *b*_*1*_ *+a*_*2*_ *b*_*2*_ *)(s*_*X*_ *)/s*_*Y*_ had comparable ranges of standardized bias. However, the *ratio* mediated had a slightly larger range of standardized bias than both (*a*_*1*_ *b*_*1*_ *+a*_*2*_ *b*_*2*_ *)/s*_*Y*_ and (*a*_*1*_ *b*_*1*_ *+a*_*2*_ *b*_*2*_ *)(s*_*X*_ *)/s*_*Y*_, and the *proportion* mediated had the largest range of standardized bias. It is interesting to note that while standardized bias ranges stayed constant across sample size for both (*a*_*1*_ *b*_*1*_ *+a*_*2*_ *b*_*2*_ *)/s*_*Y*_, (*a*_*1*_ *b*_*1*_ *+a*_*2*_ *b*_*2*_ *)(s*_*X*_ *)/s*_*Y*_, and the *ratio* mediated, standardized bias actually increased as sample size increased for the *proportion* effect size.

#### Power

Among the percentile bootstrap estimates of the four effect-size measures, (*a*_*1*_ *b*_*1*_ *+a*_*2*_ *b*_*2*_ *)/s*_*Y*_ and (*a*_*1*_ *b*_*1*_ *+a*_*2*_ *b*_*2*_ *)(s*_*X*_ *)/s*_*Y*_ had consistently higher power than the *proportion* and *ratio* mediated. When X was continuous and *c’*=0.4, the *proportion* mediated had identical power to the standardized effect-size measures at sample sizes of at least 100, while power of the *ratio* mediated was lower. For binary X, this trend occurred at sample sizes of at least 500. At N = 50 and 100 the bias-corrected bootstrap estimates of the effect size with the highest power depended on the parameter values; for the majority of parameter combinations, (*a*_*1*_ *b*_*1*_ *+a*_*2*_ *b*_*2*_ *)/s*_*Y*_ was the effect-size measure with the highest power. At N = 500 and N = 1,000 the standardized effect-size measures had the most power in the majority of parameter combinations and the *proportion* mediated often had identical power to the standardized effect-size measures, while the *ratio* mediated had less power.

#### Type I error rate

The Type I error rates for all methods were lower than 0.025 for the percentile bootstrap estimates of all four effect-size measures. The bias-corrected bootstrap estimates of the *proportion* and *ratio* mediated had high Type I error rates (equal to or above 0.07) at N = 50 and 100 when *c’*=0.131 for continuous X, and when *c’*=0.131 and 0.4 for binary X. The bias-corrected bootstrap estimates of the standardized effect-size measures had Type I error rates below 0.025 for all parameter combinations and sample sizes tested in this study.

#### Coverage

The percentile and bias-corrected bootstrap estimates of the standardized effect-size measures never had coverage below 0.925 for the effect sizes and sample sizes examined in this study. The percentile and bias-corrected bootstrap estimates of the *proportion* and *ratio* mediated had coverage below 0.925 for certain parameter combinations, however, this occurrence was less frequent for sample sizes of at least 500, for *c’* of at least 0.4, and for the percentile bootstrap intervals.

#### Interval width

**Fig. 4:**
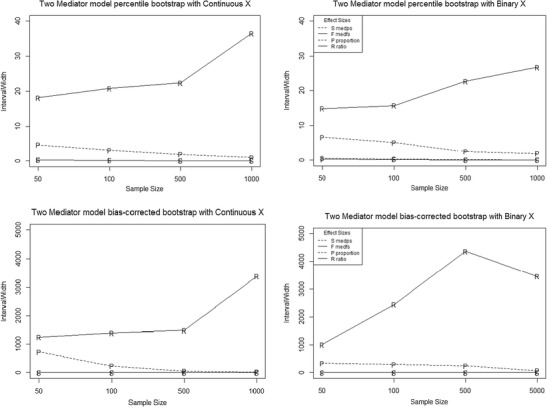
Trellis plot of interval width for percentile and bias-corrected bootstrap estimates of all effect size measures as a function of sample size for the two-mediator model. The letter markers indicate the following: S codes *med*_*ps*_, F codes *med*_*fs*_, P codes the *proportion* mediated, and R codes the *ratio* mediated

Interval widths of percentile and bias-corrected bootstrap estimates of (*a1b1+a2b2)/sY* and (*a1b1+a2b2)(sX)/sY* were consistently lower than interval widths of the *proportion* and *ratio* mediated (Fig. 4). As previously noted, the effect-size measures are on different metrics, thus making their interval widths not comparable. However, the values of interval width attained by the *ratio* mediated in certain parameter combinations were larger than 2,000, which is a striking finding even without using the interval width of other effect-size measures as a comparison.

#### Imbalance

When *c’*=0 and the *ratio* mediated was undefined, the percentile and bias-corrected bootstrap estimates of the *proportion* mediated had the highest imbalance for the majority of parameter combinations. When *c’*=0.131 the *ratio* mediated had the highest imbalance. With continuous X and for bias-corrected bootstrap estimates with binary X, as the value of the direct path and the sample size increased, the four effect-size measures all had lower imbalance and the effects size measure with the highest imbalance changed from one parameter combination to another with no discernible pattern. With binary X even at larger values of *c’* and sample size, the percentile bootstrap estimates of the *proportion* and *ratio* mediated still tended to have more imbalance than the standardized effect-size measures. When N = 100 or smaller and the direct path was small or 0, the standardized effect-size measures had lower imbalance than the *proportion* and the *ratio* mediated. If X was continuous and for binary X with the bias-corrected bootstrap, this effect dissipated with larger sample sizes and larger values of the direct path.

## Discussion

In both the single and the two-mediator models, the standardized effect-size measures had low bias and high efficiency, which was not the case for the *proportion* and *ratio* mediated. Also, the standardized effect-size measures had interval estimates with high power, no excessive Type I error rates, coverage within or above the robustness criterion, less imbalance, and lower interval width, which was not the case for interval estimates of the *proportion* and *ratio* mediated. Bias-corrected bootstrap intervals had excessive Type I error rates for certain parameter combinations, which has also been found by Fritz, Taylor, and MacKinnon (2012).

Results from all Monte Carlo studies described in this article are available online at https://figshare.com/s/88e47e000e775c455475 .

## Monte Carlo study of Bayesian estimators

Results from the Monte Carlo studies above have shown that frequentist point and interval estimates for effect-size measures for mediation models can be biased and have unsatisfactory interval properties unless the sample size and/or the effects are large. An alternative way of computing point and interval summaries for effect-size measures are to use Bayesian methods. In the frequentist framework, there is often a trade-off between the power and Type I error rate of a method, as the two vary together. This is most apparent in the case of the bias-corrected bootstrap, which is an estimator that tends to have high power, but also produces excessive Type I error rates. With Bayesian methods, one can still obtain meaningful information from a study without having to consider Type I error rate and power (van de Schoot and Depaoli 2014). In the absence of prior information, Bayesian methods for computing interval summaries of the unstandardized mediated effect have comparable statistical properties to the distribution of the product and percentile bootstrap confidence limits (Miočević, MacKinnon, & Levy, 2016). However, Bayesian methods for computing effect sizes in mediation models have not been described before, nor have the statistical properties of Bayesian point and interval summaries of effect-size measures been evaluated in Monte Carlo studies. The following paragraphs introduce Bayesian methods as an alternative to classical methods for effect size computation. This introduction is followed up by a description of a small simulation study evaluating the relative bias of Bayesian point summaries and the coverage of Bayesian interval summaries of the four effect-size measures for the parameter combinations where no frequentist estimates of effect-size measures had satisfactory relative bias. Note that this simulation study was smaller in scope than other simulation studies in this project, and was designed to probe the statistical properties of Bayesian methods for effect size computation.

The primary distinction between Bayesian and frequentist philosophies lies in their respective applications of the probability concept. In the Bayesian school of thought, probability is a measure of uncertainty (Gelman, Carlin, Stern, & Rubin, 2004), and is thus subjective. In order to reflect the uncertainty about parameters, the Bayesian framework places distributions around parameters. Thus, in the Bayesian framework, the prior information, and the final estimate are both in distribution form. The Bayes Theorem is expressed with the following formula:

16 $$ p\left(\theta \Big| data\right)=\frac{p\left(\theta, data\right)}{p(data)}=\frac{p\left(\theta \right) p\left( data\Big|\theta \right)}{p(data)} $$

where *p*(*θ*|*data*) represents the posterior distribution, *p*(*θ*) is the prior distribution placed on unknown parameters in the model, *p*(*data*|*θ*) is the likelihood function, and *p*(*data*) is a constant with respect to the parameter of interest, and can thus be omitted in order to produce a simpler way to compute a quantity proportional to the posterior distribution:

17 $$ p\left(\theta \Big| data\right)\propto p\left(\theta \right) p\left( data\Big|\theta \right) $$

In order to report a point summary for a parameter one would compute the mean, median, or mode of the posterior distribution of the given parameter. Interval estimates in the Bayesian framework are called credibility intervals, and can be either equal-tail or highest posterior density (HPD) intervals. Equal-tail credibility intervals are computed by taking the α/2th and the (1-α/2)th percentiles of the posterior distribution for a parameter. The HPD intervals are constructed based on the density of the posterior distribution, and obey the rule that no value outside the HPD interval has a higher probability than any value inside the HPD interval (Gelman, Carlin, Stern, & Rubin, 2004). Given that the distribution of the product is not symmetric and that all effect sizes are computed by multiplying parameters, the HPD intervals seem like a more promising method for summarizing the posterior distribution of the effect-size measures for the indirect effect than the equal-tail credibility intervals. The most common criticism of Bayesian methods states that the inclusion of a prior distribution in the statistical analysis introduces subjectivity that might lead the results away from reality, in the researcher’s desired direction. However, one can use non-informative prior distributions in a Bayesian analysis and thus obtain numerical estimates that are fairly similar to results from the frequentist analysis, but with different interpretations.

A simulation study was conducted to evaluate the potential of Bayesian point summaries in the single and parallel two-mediator models for parameter combinations at which frequentist methods encountered issues (i.e., excessive relative bias), while also making sure that the interval properties of credibility intervals for the effect-size measures are satisfactory. Both the point and interval properties of Bayesian summaries of the posterior distributions of effect-size measures were evaluated, and the point estimates were compared to frequentist point estimates in terms of relative bias. Combinations of parameter values for the simulation were selected based on problematic relative bias values from the Monte Carlo study of Classical Estimators at N = 50. Furthermore, the coverage of equal-tail and highest posterior density (HPD) credibility intervals of effect-size measures was evaluated.

## Method

The findings from the Monte Carlo study of Classical Estimators indicate that for the single-mediator model there were six parameter combinations with excessive relative bias when X was continuous, and 11 when X was binary, and for the two-mediator model there were 11 parameter combinations with excessive relative bias when X was continuous, and ten when X was binary. The values of parameters in these 17 combinations for the single-mediator model and the 21 combinations for the two-mediator model are summarized in Tables 1 and 2.

**Table 1:** Parameter values for the Study 3 simulation of the single-mediator model. The sample size is 50 in all conditions

	*a*	*b*
Continuous X		
c’=0	0.14	0.14
c’=0.14	0.14	0.39
c’=0.39	0.14	0.14
	0.14	0.59
	0.39	0.39
c’=0.59	0.14	0.59
Binary X		
c’=0	0.14	0.39
	0.39	0.39
c’=0.14	0.14	0.14
	0.14	0.39
	0.14	0.59
	0.39	0.14
c’=0.39	0.14	0.39
	0.39	0.14
	0.59	0.14
c’=0.59	0.14	0.39
	0.39	0.59

**Table 2:** Parameter values for the Study 3 simulation of the parallel two-mediator model. The sample size is 50 in all conditions

	*a*_*1*_ *=b*_*1*_	*a*_*2*_ *=b*_*2*_
Continuous X		
c’=0	0	0.101
	0.101	0
	0.101	0.101
c’=0.131	0	0.314
	0.101	0
c’=.4	0	0.101
	0.101	0
c’=0.74	0	0.101
	0.101	0
	0.101	0.101
	0.314	0.101
Binary X		
c’=0	0	0.101
	0.101	0
	0.314	0.101
c’=0.131	0.101	0
c’=0.4	0	0.101
	0.101	0
	0.101	0.101
c’=0.74	0	0.101
	0.101	0
	0.101	0.101

SAS software (Version 9.3 of the SAS System for Windows) was used to conduct a simulation which calculated relative bias of the mean and median of the posterior distributions of *ab*_*ps*_, *ab*_*fs*_, the *proportion* and the *ratio* mediated at N = 50 for 17 combinations of population values for *a*, *b*, and *c’* paths, and 21 combinations of population values of *a1, a2, b1, b2*, and *c’* paths. Equal-tail and HPD intervals were computed for each iteration and coverage of both types of credibility intervals was computed over 1,000 iterations. The adequacy of coverage was assessed using Bradley’s robustness criterion (1978), also used in the Monte Carlo study of Classical Estimators. The prior distributions for all of the parameters (coefficients *a, b, c’,* error variances of M and Y, in the single-mediator model, and *a1, a2, b1, b2*, and *c’*, and error variances of M_1_, M_2_, and Y in the two-mediator model) were diffuse, as in Miočević and MacKinnon (2014). Regression coefficients were assigned normal priors with a mean hyperparameter equal to 0, and a precision hyperparameter of 10^-3^. Residual variances were assigned inverse gamma priors with the shape and inverse scale (denoted iscale in SAS PROC MCMC) hyperparameters equal to 0.01. For more on principles and applications of Bayesian mediation analysis, see Yuan and MacKinnon (2009), Enders, Fairchild, and MacKinnon (2014), and Miočević and MacKinnon (2014). Example SAS code for all Monte Carlo studies in this manuscript is available online at https://figshare.com/s/8d48fed4a23fff78e2a3 .

## Results

In the single-mediator model, a comparison between the relative bias of frequentist estimates and Bayesian point summaries (mean and median) indicated that on average the mean of the posterior for the four effect-size measures had slightly larger average relative bias than the frequentist estimate, and the median of the posterior had lower average relative bias than the frequentist estimate. The reduction in relative bias in the posterior median relative to the frequentist estimate was most pronounced for the *proportion* mediated, followed closely by the *ratio* mediated. For the standardized effect-size measures the mean of the posterior had up to .13 higher average relative bias than the corresponding frequentist estimate, while the median of the posterior had lower average relative bias than the frequentist estimate in the majority of parameter combinations. Thus, in the single mediator model the median of the posterior of an effect-size measure is preferred over the frequentist estimate and the mean of the posterior in terms of relative bias for the parameter combinations in this study.

In the two-mediator model the average relative bias of the median of the posteriors for the standardized effect measures was lower than the relative bias of the corresponding frequentist estimate for all parameter combinations in this study. The relative bias of the mean of the posterior for the standard effect-size measures had lower average relative bias than the frequentist estimator in some parameter combinations, however, as was found for the single-mediator model, in the two-mediator model the posterior median was a better choice than the posterior mean in terms of relative bias. The average relative bias of the median of the *proportion* and the *ratio* mediated was lower than the relative bias of the corresponding frequentist estimate in the majority of the parameter combinations. In all, the median of the posterior emerged as a point summary with less relative bias than the mean of the posterior, and in the majority of parameter combinations in this study the median of the posterior also had lower average relative bias than the corresponding frequentist estimate of a given effect-size measure.

In addition to point summaries of the posterior distributions of the effect-size measures, the coverage of equal-tail and highest posterior density intervals for the same parameter combinations was also evaluated. For the single-mediator model the coverage of equal-tail credibility intervals was usually within 0.025 of the nominal value of 0.95, and for parameter combinations for which this was not the case, coverage was above 0.975. Highest posterior density intervals had more instances of coverage above 0.975 than equal-tail credibility intervals. In the two-mediator model the majority of parameter combinations had coverage above 0.975 for all effect-size measures, except in a few instances where the *proportion* mediated had coverage below 0.925. The *proportion* mediated is the only effect-size measure that had instances of coverage below 0.925 with both equal-tail and highest posterior density intervals in both the single and the two-mediator model. Results from all Monte Carlo studies in this project are available online at https://figshare.com/s/88e47e000e775c455475 .

## Discussion

Overall, Bayesian methods seem to be a promising new way to reduce relative bias of effect-size measures for the indirect effect, while also maintaining some desirable interval properties. The findings of this small simulation study with N = 50 indicate that while Bayesian methods did not produce point summaries of the *proportion* and the *ratio* mediated with satisfactory relative bias in the single-mediator model, there is evidence that Bayesian methods can reduce the relative bias of the *proportion* and the *ratio* mediated in the parallel two-mediator model. Future work should address how including prior information could be used to improve Bayesian estimation of the effect-size measures.

## Empirical examples

The following examples illustrate the kinds of interpretations that can be made using the effect sizes examined in this article. The data for these empirical examples come from a prevention study of anabolic steroid use among adolescents (Goldberg et al., 1996; MacKinnon et al., 2001). The sample for the analysis below consisted of 1,315 high school football players, 46% of whom were in the treatment condition and received a 14-session prevention program, and the remaining 54% of participants were in the control condition and received a pamphlet on steroid use. The outcome of interest for the empirical examples below was participants’ training self-efficacy, and because the outcome was continuous and not measured in units that are readily interpretable, several effect sizes were computed for the indirect effect.

### Single-mediator model

For the single-mediator model, the indirect effect of treatment (X) on training self-efficacy (Y) through team as an information source (M) was considered. The observed values of *a, b, c,* and *c’* were 0.549, 0.174, 0.318, and 0.234 (respectively), and the dependent variable Y had a standard deviation of 1.217. The indirect effect was statistically significant and equal to 0.091, meaning that being in the treatment group (X) resulted in a 0.091 point increase in training self-efficacy (Y) through team as an information source (M).

Point estimates, point summaries of the posterior distribution, percentile and bias-corrected bootstrap limits, as well as equal-tail and highest posterior density (HPD) intervals of effect-size measures for the indirect effect (Table 3) were computed using SAS System Version 9.3 for Windows. The fully standardized indirect effect was not computed because X is binary, and thus the partially standardized indirect effect has a more intuitive interpretation. These estimates indicated that being in the treatment group resulted in an increase of 0.078 (or 0.081 and 0.80, if one chooses to report the mean and the median, respectively, of the posterior distribution for the partially standardized indirect effect) standard deviations in training self-efficacy through team as an information source. Also, 29% (or 35% and 30.3%, according to the mean and median of the posterior distribution of the *proportion* mediated, respectively) of the effect of treatment condition on training self-efficacy was mediated by team as information source, and the indirect effect in this model was 0.41 (or 0.72 and 0.43, according to the mean and median of the posterior distribution of the *ratio* mediated) times the size of the direct effect.

**Table 3:** Effect sizes for the indirect effect from group (X) on training self-efficacy (Y) through team as information source (M)

Effect size	Point estimates freq post mean post median	Percentile bootstrap	Bias-corrected bootstrap	Bayesian equal-tail credibility intervals	Bayesian HPD credibility intervals
ab_ps_	0.078 0.081 0.080	[0.028, 0.135]	[0.028, 0.135]	[0.037, 0.135]	[0.033, 0.129]
Proportion	0.290 0.350 0.303	[0.103, 0.789]	[0.116, 0.870]	[0.139, 0.789]	[0.122, 0.678]
Ratio	0.408 0.719 0.435	[0.085, 2.550]	[0.091, 2.727]	[0.151, 3.070]	[0.122, 2.026]

*freq* frequentist estimate, *post mean* posterior mean, *post median* posterior median, *HPD* highest posterior density

The interval estimates (and summaries) for the three effect-size measures in the single-mediator model were consistent with the conclusion of the significance test for the indirect effect in that none of the intervals contained zero. Given the results of the simulation, all three effect sizes had bootstrap intervals with satisfactory statistical properties, and thus all could be reported. However, the intervals for the *ratio* mediated have a somewhat confusing interpretation, given that the intervals indicate that it is possible that the indirect effect is less than 10% of the direct effect, and also more than twice as large as the direct effect. This is an example of a situation where the interval for the *ratio* mediated communicates that it is possible that the indirect effect was smaller, equal to, or larger than the direct effect.

### Two-mediator model

For the case of a binary independent variable with two parallel continuous mediators and one continuous outcome, the example above can also be used to compute multiple effect sizes. The multiple mediator hypothesis of the experiment was that the effect of group (X) on training self-efficacy (Y) would be mediated by team as an information source (M_1_) as well as perceived severity of steroid use (M_2_). The observed values of *a*_*1*_ *, b*_*1*_ *, a*_*2*_ *, b*_*2*_ *, c,* and *c’* were 0.549, 0.165, 0.440, 0.092, 0.318, and 0.188 (respectively), and the dependent variable Y had a standard deviation of 1.217. The indirect effect *a*_*1*_ *b*_*1*_ *+a*_*2*_ *b*_*2*_ *=*0.131 was statistically significant, as determined by the percentile bootstrap intervals of the total indirect effect. Point estimates, point summaries of the posterior distribution, percentile and bias-corrected bootstrap interval estimates, and equal-tail and HPD intervals of three effect-size measures were obtained using SAS System Version 9.3 for Windows (Table 4). The fully standardized indirect effect was not computed because X is binary, and the partially standardized indirect effect is more interpretable in this situation.

**Table 4:** Effect sizes for the indirect effect from group (X) on training self-efficacy (Y) through team as information source (M_1_) and perceived severity of steroid use (M_2_)

Effect size	Point estimate freq post mean post median	Percentile bootstrap	Bias-corrected bootstrap	Bayesian equal-tail credibility intervals	Bayesian HPD credibility intervals
Med_ps_	0.108 0.105 0.106	[0.059, 0.183]	[0.046, 0.161]	[0.054, 0.154]	[0.052, 0.151]
Proportion	0.411 0.412 0.378	[0.193, 1.055]	[0.190, 0.950]	[0.163, 0.853]	[0.153, 0.734]
Ratio	0.697 −0.526 0.598	[−1.986, 4.915]	[0.137, 5.213]	[0.186, 3.308]	[0.133, 2.484]

*freq* frequentist estimate, *post mean* posterior mean, *post median* posterior median, *HPD* highest posterior density

The treatment increased training self-efficacy by 0.108 (0.105, according to the mean, and 0.106 according to the median of the posterior distribution of *ab*_*ps*_) standard deviations through team as information source (M_1_) and perceived severity of steroid use (M_2_). The total indirect effect through team as an information source and perceived severity of steroid use was 41.1% (or 41.2% according to the mean, and 37.8% according to the median of the posterior distribution of the *proportion* mediated) of the total effect of treatment on training self-efficacy. The total indirect effect was 0.697 (or 0.526 according to the mean, and 0.598 according to the median of the posterior distribution of the *ratio* mediated) times the size of the direct effect of treatment on training self-efficacy (Table 4).

Not all interval estimates for the three effect-size measures in the parallel two-mediator model were consistent with the conclusion of the significance test for the indirect effect. The percentile bootstrap interval for the *ratio* mediated contained zero. Furthermore, the fact that the percentile bootstrap interval for the *proportion* mediated had an upper limit greater than 1 illustrates a case when the proportion mediated is not bounded, and thus can have a non-intuitive interpretation of the indirect effect being more than 100% of the total effect. Both the percentile and bias-corrected bootstrap confidence intervals for the *ratio* mediated had lower limits below 1 and upper limits above 1, which is an example of a case where the mediated effect could be a fraction of the direct effect, equal to the direct effect, and several times larger than the direct effect.

The above empirical example illustrates both the usefulness of the standardized effect-size measures in a situation where the outcome is in units that are not easy to interpret, and the issues associated with interpreting the *proportion* and *ratio* mediated when their interval estimates are excessively wide.

## General discussion

### Summary of findings

In the single-mediator model, *ab/s*_*Y*_ and *ab(s*_*X*_ *)/s*_*Y*_ have satisfactory relative bias levels, whereas the *proportion* and *ratio* mediated have large relative bias in the majority of combinations of sample size and parameter values. For some parameter combinations at which no effect-size measures in the Monte Carlo study of classical estimators had relative bias below .05, Bayesian methods offered point summaries with satisfactory relative bias. The stability of the four effect-size measures in the single-mediator model depends on the size of the coefficients and sample size.

In the two-mediator model, the standardized effect-size measures have lower relative bias than the *proportion* and *ratio* mediated. Effect sizes (*a*_*1*_ *b*_*1*_ *+a*_*2*_ *b*_*2*_ *)/s*_*Y*_ and (*a*_*1*_ *b*_*1*_ *+a*_*2*_ *b*_*2*_ *)(s*_*X*_ *)/s*_*Y*_ were found to be efficient at all sample sizes, and become more efficient as sample size increases, whereas efficiency of the *proportion* and *ratio* mediated effect sizes does not change in a predictable manner with increased sample size. The percentile and bias-corrected bootstrap had satisfactory interval properties for standardized effect-size measures, and Bayesian equal-tail and highest posterior density credibility intervals emerged as promising alternatives for interval computation. Findings from prior literature were supported in that the bias-corrected bootstrap intervals had excessive Type I error rates in some situations (Fritz, Taylor, & MacKinnon, 2012), and in that bootstrap methods had satisfactory coverage for the fully standardized indirect effect (Cheung, 2009).

### Further considerations

Ideally, a researcher will choose a meaningful effect size with the least bias and most stability. In light of these findings, a general recommendation for the single-mediator model with continuous X would be to choose either *ab/sY* or *ab(sX)/sY*, and to use *(a1b1+a2b2)/sY* or *(a1b1+a2b2)(sX)/sY* for the two-mediator model. In the single-mediator model with binary X, one would opt for *ab/sY*, and *(a1b1+a2b2)/sY* is recommended for the two-mediator model. Although *ab(sX)/sY* and *(a1b1+a2b2)(sX)/sY* performed adequately, the fully standardized effect size is based on change for a standard deviation in X and so for a study with binary X, the fully standardized effect size would not offer a more intuitive interpretation than the raw indirect effect or the other effect sizes in this study (Hayes, 2013; MacKinnon, 2008). This situation highlights the importance of interpretation for the selection of an effect-size measure. When choosing an effect-size measure to represent the indirect effect, one should pick the effect-size measure that answers the research question most accurately. If one is interested in the standardized change produced in Y by X through M, then either *ab/s*_*Y*_ (for situations where X is binary or has an easily interpretable scale) or *ab(s*_*X*_ *)/s*_*Y*_ (for cases where X is continuous and has a scale that is not intuitive to the reader) are ideal.

Some authors have criticized standardized effect-size measures for their dependence on factors that influence the variance of the sample, such as study design, sampling strategy, and choice of covariates (Greenland, Schlesselman, & Criqui, 1986). It was later pointed out that standardized coefficients could be useful in comparing effects of one variable in different studies if the compared coefficients are adjusted for the same covariates, if the variable is normally distributed, and if a common multiplier and pooled standard deviation are used to standardize all effects that are being compared (Greenland, Maclure, Schlesselman, Poole, & Morgenstern, 1991). The *proportion* and *ratio* mediated have clear and useful interpretations, however, given their bias and instability, the *proportion* mediated is not a good choice unless sample size is above 500 (for continuous X), and the *ratio* mediated is a poor choice unless sample size is above 2,000 (for continuous X), or above 5,000 (for binary X) (MacKinnon, Warsi & Dwyer, 1995).

If one wishes to plan the sample size of a study based on the availability of unbiased and efficient effect-size measures for the indirect effect and the research question can only be answered with the *ratio* mediated, one should be mindful of the expected size of *c’* since this effect-size measure is only unbiased for *c’≥*0.39 and sample sizes of at least 500. Effect sizes are generally computed after the mediation analysis, thus the sizes of the estimates are known. The findings from these studies provide guidelines for the optimal effect size for a given value of sample size; however, one should always keep the meaning and interpretation of these effect-size measures in mind. Another potential application of the findings from this study is meta-analyses: summarizing the findings of numerous mediation studies may require converting all the effect sizes into the same metric, one that is least biased and most stable.

Regarding software, all effect-size measures, confidence intervals using percentile and bias-corrected bootstrap from this study for the single and two-mediator models can be computed using PROCESS (Hayes, 2013). Furthermore, the code for obtaining frequentist point estimates, bootstrap confidence limits, and Bayesian point and interval summaries of effect-size measures in this study is also available from the first author upon request. All effect-size measures for the single-mediator model can be computed using the *mediate* function in the R package MBESS (Kelley, 2007a, b). All effect-size measures in this paper can also be computed using Mplus, and it is possible to obtain bootstrap estimates as well as point summaries and credibility intervals using the ESTIMATOR=BAYES option. There are two ways to obtain the standardized effect-size measures in Mplus; one is to compute the indirect effect and ask for STDY and STDYX standardization, and the second way would be to label the variances of X and Y and use these values in the MODEL CONSTRAINT statement to obtain *ab/s*_*Y*_, *ab(s*_*X*_ *)/s*_*Y*_ for the single-mediator model and *(a1b1+a2b2)/sY* and *(a1b1+a2b2)(sX)/sY* for the two-mediator model.

Analytic solutions for standard errors of each effect size for the mediation effect may confirm and explain findings from simulation studies and strengthen recommendations about the usefulness of particular effect-size measures in meta-analyses of mediation models. However, confidence intervals based on an assumed normal distribution for an effect-size measure and the corresponding analytic formula for the standard error of the effect-size measure may not be accurate, thus making the bootstrap method ideal. Like normal theory confidence limits, interval estimates obtained using the bootstrap methods still have an interpretation in terms of repeated sampling, and in order to interpret results in terms of probability one needs to use Bayesian methods to construct credibility intervals for effect-size measures (Miočević & MacKinnon, 2014; Yuan & MacKinnon, 2009). To the best of our knowledge, this project contains the first study that evaluates the usefulness of Bayesian methods in effect size computation, and possible extensions of this line of research are to evaluate different priors from the ones considered in this study. It is also important for future research to examine whether effect-size measures are unbiased and efficient with more than two mediators, in multilevel mediation models (Stapleton, Pituch & Dion, 2014), and in path analysis models.

In summary, this research and prior studies point to the standardized effect sizes as the best mediation measures. Prior research has demonstrated that for individual paths in the mediated effect, correlations and standardized path measures are generally unbiased and accurate (Fairchild et al., 2009; Taborga, 2000). It is important to keep in mind that other proposed promising effect-size measures for the entire mediation effect also have limitations such as the instability of the *ratio* and *proportion* mediated (MacKinnon, Warsi & Dwyer, 1995; MacKinnon, 2008), the possibility of negative and non-intuitive values for R^2^ (de Heus, 2012; Fairchild et al., 2009) and most recently the lack of monotonicity for *κ* ^2^ (Wen & Fan, 2015). As a result, it is important to consider the possible limitations of the standardized indirect effect-size measures. As mentioned above, one limitation of the standardized effect-size measures is either restricted or excessive variability in Y, and also X if the fully standardized measure is used. However, this limitation is also present for simpler effect-size measures such as the *d* effect-size measure for the difference between two independent groups. In addition, there are not yet guidelines for small, medium, and large standardized indirect effects though links with literature on the *d* effect size may shed light on this topic. Perhaps the usefulness of these new effect-size measures is best evaluated by application to actual research data. In this paper, the standardized effect-size measures for the indirect effect are generally unbiased in single and multiple mediator models, have a clear interpretation, and can be extended to more complicated models.

## References

[CR1] Wilkinson L, American Psychological Association Task Force on Statistical Inference (1999). Statistical methods in psychology journals: Guidelines and explanations. American Psychologist.

[CR2] Barreto M, Ellemers N (2005). The burden of benevolent sexism: How it contributes to the maintenance of gender inequalities. European Journal of Social Psychology.

[CR3] Biesanz JC, Falk CF, Savalei V (2010). Assessing mediational models: Testing and interval estimation for indirect effects. Multivariate Behavioral Research.

[CR4] Bradley JV (1978). Robustness?. British Journal of Mathematical and Statistical Psychology.

[CR5] Chassin L, Pitts SC, DeLucia C, Todd M (1999). A longitudinal study of children of alcoholics: Predicting young adult substance use disorders, anxiety, and depression. Journal of Abnormal Psychology.

[CR6] Cheung MW (2007). Comparison of approaches to constructing confidence intervals for mediating effects using structural equation models. Structural Equation Modeling: A Multidisciplinary Journal.

[CR7] Cheung MW (2009). Comparison of methods for constructing confidence intervals of standardized indirect effects. Behavior Research Methods.

[CR8] Cohen J (1988). Statistical power analysis for the behavioral sciences. Hillsdale, NJ: Lawrence Erlbaum Associates.De Heus, P. (2012). R squared effect-size measures and overlap between direct and indirect effect in mediation analysis. Behavior Research Methods.

[CR9] Craig, C. C. (1936) On the Frequency Function of xy.* The Annals of Mathematical Statistics 7*(1), 1–15

[CR10] Enders CK, Fairchild AJ, MacKinnon DP (2013). A Bayesian approach for estimating mediation effects with missing data. Multivariate Behavioral Research.

[CR11] Fairchild AJ, MacKinnon DP, Taborga MP, Taylor AB (2009). R^2^ effect-size measures for mediation analysis. Behavior Research Methods.

[CR12] Feingold, A. (2014). Confidence Interval Estimation for Standardized Effect Sizes in Multilevel and Latent Growth Modeling. *Journal of Consulting and Clinical Psychology*, 1–12. 10.1037/a0037721 PMC4324017 25181028

[CR13] Freedman LS (2001). Confidence intervals and statistical power of the ‘validation’ ratio for surrogate or intermediate endpoints. Journal of Statistical Planning and Inference.

[CR14] Fritz MS, Taylor AB, MacKinnon DP (2012). Explanation of two anomalous results in statistical mediation analysis. Multivariate Behavioral Research.

[CR15] Gelman A, Carlin JB, Stern HS, Rubin DB (2004). Bayesian data analysis.

[CR16] Goldberg, L., Elliot, D. L., Clarke, G. N., MacKinnon, D. P., Zoref, L., Moe, E., … & Wolf, S. L. (1996). The Adolescents Training and Learning to Avoid Steroids (ATLAS) prevention program: background and results of a model intervention. *Archives of pediatrics & adolescent medicine*, *150*(7), 713–721. 10.1001/archpedi.1996.02170320059010 8673196

[CR17] Greenland S, Maclure M, Schlesselman JJ, Poole C, Morgenstern H (1991). Standardized regression coefficients: a further critique and review of some alternatives. Epidemiology.

[CR18] Greenland S, Schlesselman JJ, Criqui MH (1986). The fallacy of employing standardized regression coefficients and correlations as measures of effect. American Journal of Epidemiology.

[CR19] Hayes A (2013). Miscellaneous Topics in Mediation Analysis. Mediation, Moderation, and Conditional Process Analysis.

[CR20] Ilies R, Judge TA (2003). On the heritability of job satisfaction: the mediating role of personality. Journal of Applied Psychology.

[CR21] Ilies R, Judge TA (2005). Goal regulation across time: The effects of feedback and affect. Journal of Applied Psychology.

[CR22] Kaplan D (1988). The impact of specification error on the estimation, testing, and improvement of Structural Equation Models. Multivariate Behavioral Research.

[CR23] Kelley K (2005). The effects of nonnormal distributions on confidence intervals around the standardized mean difference: Bootstrap and parametric confidence intervals. Educational and Psychological Measurement.

[CR24] Kelley, K. (2007a). Confidence intervals for standardized effect sizes: Theory, application, and implementation. *Journal of Statistical Software, 20*(8), 1-24.

[CR25] Kelley K (2007). Methods for the behavioral, educational, and social sciences: An R package. Behavior Research Methods.

[CR26] Kraemer HC (2014). A mediator effect size in randomized clinical trials. International Journal of Methods in Psychiatric Research.

[CR27] Krull JL, MacKinnon DP (1999). Multilevel mediation modeling in group-based intervention studies. Evaluation Review.

[CR28] Lee SY, Song XY (2004). Evaluation of the Bayesian and maximum likelihood approaches in analyzing structural equation models with small sample sizes. Multivariate Behavioral Research.

[CR29] Leigh JP (1983). Direct and indirect effects of education on health. Social Science & Medicine.

[CR30] MacKinnon DP (2008). Introduction to statistical mediation analysis.

[CR31] MacKinnon, D. P., Goldberg, L., Clarke, G. N., Elliot, D. L., Cheong, J., Lapin, A., … & Krull, J. L. (2001). Mediating mechanisms in a program to reduce intentions to use anabolic steroids and improve exercise self-efficacy and dietary behavior. *Prevention Science, 2*(1), 15–28. 10.1023/a:1010082828000 11519372

[CR32] MacKinnon DP, Johnson CA, Pentz MA, Dwyer JH, Hansen WB, Flay BR, Wang EY (1991). Mediating mechanisms in a school-based drug prevention program: First-year effects of the Midwestern Prevention project. Health Psychology.

[CR33] MacKinnon DP, Warsi G, Dwyer JH (1995). A simulation study of mediated effect measures. Multivariate Behavioral Research.

[CR34] Manly, B. F. (1997). Randomization, bootstrap and Monte Carlo methods in Biology. Cornwall: T.J. International Ltd.

[CR35] Miočević, M., & MacKinnon, D. P. (2014). SAS® for Bayesian Mediation Analysis. *In Proceedings of the SAS Global Forum 2014 Conference, Cary NC: SAS Institute Inc.*

[CR36] Miočević, M., MacKinnon, D. P., & Levy, R. (2016). *Comparison of Bayesian and frequentist estimates of the mediated effect*. Manuscript under review.

[CR37] O’Rourke HP, MacKinnon DP (2015). When the test of mediation is more powerful than the test of the total effect. Behavior Research Methods.

[CR38] Preacher KJ, Kelley K (2011). Effect-size measures for mediation models: Quantitative strategies for communicating indirect effects. Psychological Methods.

[CR39] Sharkansky EJ, King DW, King LA, Wolfe J, Erickson DJ, Stokes LR (2000). Coping with Gulf War combat stress: Mediating and moderating effects. Journal of Abnormal Psychology.

[CR40] Shrout, P. E., & Bolger, N. (2002). Mediation in experimental and nonexperimental studies: New procedures and recommendations. *Psychological methods, 7*(4), 422–445. 12530702

[CR41] Stapleton LM, Pituch KA, Dion E (2015). Standardized Effect-size measures for Mediation Analysis in Cluster-Randomized Trials. The Journal of Experimental Education.

[CR42] Stice E (2001). A prospective test of the dual-pathway model of bulimic pathology: Mediating effects of dieting and negative affect. Journal of Abnormal Psychology.

[CR43] Taborga, M. P. (2000). *Effect size in mediation models*. Unpublished master’s thesis, Arizona State University, Tempe, Arizona.

[CR44] Tein JY, Sandler IN, Ayers TS, Wolchik SA (2006). Mediation of the effects of the family bereavement program on mental health problems of bereaved children and adolescents. Prevention Science.

[CR45] van de Schoot R, Depaoli S (2014). Bayesian analyses: Where to start and what to report. European Health Psychologist.

[CR46] Vacha-Haase T, Thompson B (2004). How to estimate and interpret various effect sizes. Journal of Counseling Psychology.

[CR47] Wen Z, Fan X (2015). Monotonicity of effect sizes: Questioning kappa-squared as mediation effect-size measure. Psychological Methods.

[CR48] Wolchik, S. A., West, S. G., Westover, S., Sandler, I. N., Martin, A., Lustig, J., Tein, J., & Fisher, J. (1993). The children of divorce parenting intervention: Outcome evaluation of an empirically based program. *American Journal of Community Psychology, 21(3),* 293– 331. 10.1007/BF00941505 8311029

[CR49] Yuan K-H, Chan W (2011). Biases and standard errors of standardized regression coefficients. Psychometrika.

[CR50] Yuan Y, MacKinnon DP (2009). Bayesian mediation analysis. Psychological Methods.

